# A Pathogenic Germline BRCA1 Variant in a Patient With Cellular Congenital Mesoblastic Nephroma: A Case Report

**DOI:** 10.7759/cureus.43857

**Published:** 2023-08-21

**Authors:** Gayatri Dholakia, Julia Meade

**Affiliations:** 1 Public Health, University of Pittsburgh, Pittsburgh, USA; 2 Pediatrics, University of Pittsburgh Medical Center, Pittsburgh, USA

**Keywords:** case report, genetic syndromes, pediatric hematology-oncology, brca1 germline mutation, cystic nephroma

## Abstract

Benign cystic tumors of the kidney are well-described in infants and young children. Here we report an infant diagnosed with a cellular congenital mesoblastic nephroma (CMN) with a germline pathogenic variant in BRCA1. This finding is novel because BRCA1 is an adult-onset cancer predisposition gene causing breast, ovarian, pancreatic, and prostate cancers. However, increasing studies are indicating the presence of germline BRCA1 in both malignant and benign childhood cancers.

## Introduction

Congenital mesoblastic nephromas (CMN) are the most common renal tumors in infancy [1]. Though they are pathologically distinct from other benign kidney tumors by the presence of an ETV6 fusion, they have not been associated with a germline cancer predisposition syndrome [2]. By contrast, cystic nephroma is well-associated with DICER1 syndrome, and genetic testing is recommended for any child with this diagnosis [3,4]. With a lack of associated cancer predisposition syndrome and clear guidelines for germline genetic testing in infants with CMN, panels limited to renal/urinary tract cancers, Wilms tumor or DICER1 may be used if genetic testing is taken out of context of the family history. Here, we report an infant with a CMN and a germline BRCA1 pathogenic variant, discovered via a comprehensive germline panel. 

## Case presentation

**Figure 1:**
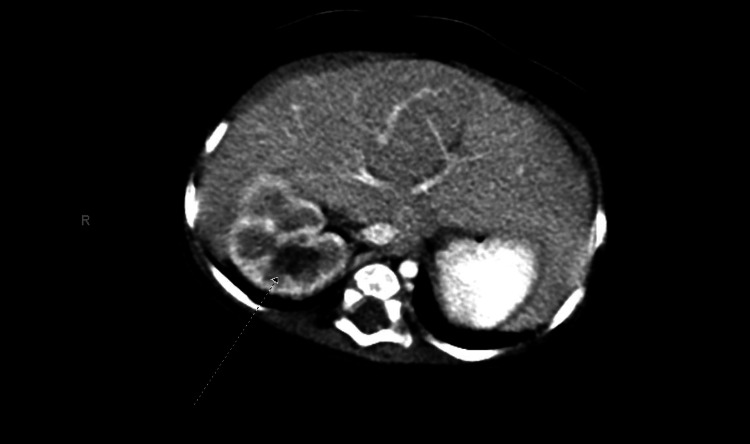
Right kidney cystic nephroma

A five-month-old boy presented with a three-day history of hematuria and blood clots in his diaper. The physical examination revealed a palpable right-sided abdominal mass and an atrophic right testis was also incidentally noted. An ultrasound revealed an 8.3 cm solid mass in the right kidney, which leads to a CT scan revealing a large, well-defined hypodense mass measuring 9.8 × 7.9 × 7.8 cm with a volume of about 302 cm^3^ arising from the inferior pole of the right kidney (Figure 1). Imaging demonstrated heterogeneous enhancement with areas of necrosis, and a claw sign on the mass suggested its origin from the kidney. The child underwent a right radical nephrectomy and right orchiectomy. The pathology report returned as a stage II cellular CMN. A t(12;15)(p13;q26) translocation was consistent with an ETV6 fusion, though the partner gene was not identified by Fluorescence in-situ hybridization (FISH). Following surgery, no further adjuvant therapy was required. The child did well with no evidence of tumor recurrence, now seven years out from surgery.

**Figure 2:**
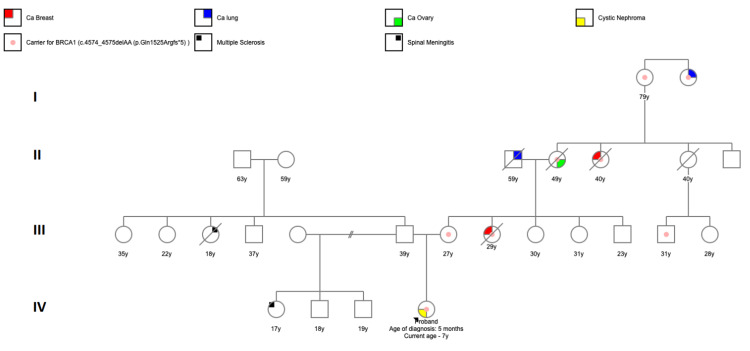
Family Pedigree

The patient was referred for a cancer predisposition evaluation due to his personal diagnosis of CMN of the kidney and strong family history of cancer on his maternal side. The family history was significant with a maternal aunt diagnosed at age 25 with stage IV breast cancer and a maternal grandmother with stage IV ovarian cancer at age 49. The maternal grandmother's sister was also diagnosed with breast cancer in her 30s (Figure 2). There was no family history of renal abnormalities.

Due to the maternal aunt’s early diagnosis of breast cancer, which occurred at the same time as the patient’s CMN diagnosis, she had undergone germline genetic testing and was known to carry a BRCA1 pathogenic variant. Based on parental preference for comprehensive germline testing, genetic testing consisting of a panel of 43 genes with a focus on solid tumors was performed. Germline genetic testing revealed a c.4574_4575delAA (p.Gln1525Argfs*5) BRCA1 pathogenic variant in our patient, giving the child a diagnosis of hereditary breast and ovarian cancer (HBOC) syndrome. The boy’s pathogenic variant was consistent with the maternal aunt’s variant and the child’s mother was counseled that she also has HBOC. This particular variant has a 40-87% risk for breast cancer, along with a male breast cancer risk of 1-2%. Screening guidelines for children do not exist, therefore no additional surveillance outside of monitoring for tumor recurrence was performed [5,6]. However, the patient will become eligible for screening guidelines for males with HBOC in adulthood. The patient's mother was referred for clinical management per the National Comprehensive Cancer Network (NCCN) guidelines. 

## Discussion

Ten percent of children with malignancy have a genetic predisposition to the development of cancer [7]. Indications of a cancer predisposition syndrome include phenotypic physical features, a family history suggestive of a cancer predisposition syndrome, specific malignancies like choroid plexus carcinoma suggestive of Li-Fraumeni syndrome (LFS), specific somatic markers of the malignancy like low-hypodiploidy in acute lymphoblastic leukemia in LFS, bilateral, metachronous, synchronous, or multiple primary tumors, and severe toxic side-effects when exposed to chemotherapy or radiation therapy as part of the routine treatment [8]. However, the absence of any of these indications does not exclude the possibility of a cancer predisposition syndrome due to the occurrence of de novo mutations, low penetrance of certain cancer predisposition syndromes, and incidental findings [9]. This case demonstrates that incidental findings on broader panel testing still have health implications for patients and their families.

A recent study by Kratz et al. reported increasing associations between adult-onset genes like BRCA1 and the development of childhood malignancies [10]. Kratz et al. conducted a meta-analysis of 11 studies that included comprehensive germline testing for children and adolescents with cancer and have established pathogenic variants in BRCA1 and BRCA2 in children with a diagnosis of acute lymphoblastic leukemia, ependymoma, Ewing sarcoma, gliomas, Langerhans cell histiocytosis, medulloblastoma, neuroblastoma, rhabdoid tumor, and rhabdomyosarcoma [10]. The dataset, however, did not include benign tumors such as cystic nephroma, which may therefore be underrepresented in the literature.

Cancer predisposition genes have historically been classified as pediatric and adult-onset cancer predisposition genes because they confer an increased risk of developing malignancies in childhood and adulthood, respectively, and the risk of cancer in the other age group is relatively low. As HBOC confers a high risk for breast cancer, ovarian cancer, pancreatic cancer, prostate cancer, and male breast cancer in adults, many providers are reticent to perform testing for this condition in the pediatric setting. However, based on parental preference and family history this genetic testing was able to impact health decision-making for the child’s family. This child is also able to receive graduated education on HBOC in annual visits to the cancer predisposition program to increase health literacy and ability to navigate cancer screening as a young adult.

## Conclusions

This case highlights the importance of assessing for germline variants in genes other than pediatric kidney tumor-related genes, most commonly DICER1, which has a well-established association with pediatric renal tumors. Patients may benefit from broader panel testing, exome, and paired tumor-germline DNA analyses to further aid the discovery of both adult and pediatric-onset cancer predisposition syndromes in rare pediatric tumors. 
